# Supplementary material for: The impact of COVID-19 vaccination campaigns accounting for antibody-dependent enhancement
Source: PLoS One. 2021 Apr 22;16(4):e0245417. doi: 10.1371/journal.pone.0245417 (PMC8061987; doi:10.1371/journal.pone.0245417)

$$B = f_{\text{Sick}} + (1 - f_{\text{Sick}}) f_I^{(\text{U},+)}$$

$$\tilde{B} = 1 - B = 1 - f_{\text{Sick}} - (1 - f_{\text{Sick}}) f_I^{(\text{U},+)}$$

$$A = f_{\text{Sick}} \; f_{\text{Dead}}$$

$$\tilde{A} = 1 - A$$

$$A^{(e)} = f_{\text{Sick}}^{(e)} \; f_{\text{Dead}}^{(e)} \qquad \text{for } e = \text{"V", "NI", "PI", "ADE", "}I,*\text{", "}L,*\text{"}$$

$$\tilde{A}^{(e)} = 1 - A^{(e)}$$

$$① = \delta \; f_{\text{Sick}}^{(\text{U},+)} f_{\text{Dead}} \qquad ② = \delta \left(1 - f_{\text{Sick}}^{(\text{U},+)} f_{\text{Dead}}\right)$$

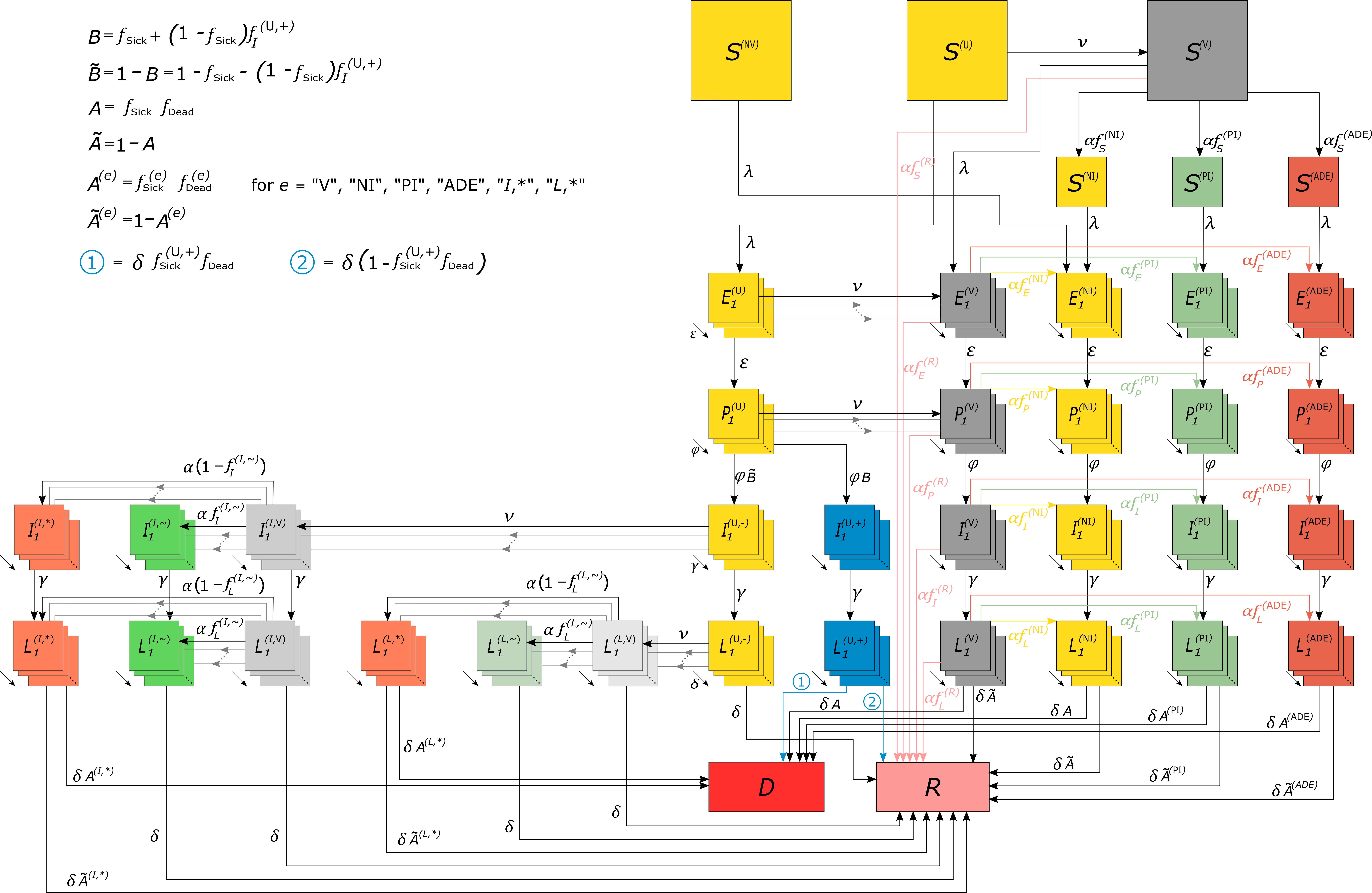

Supplement: S1 Fig — Illustration of the full model flow chart, showing all compartments (boxes) and possible transitions from compartments (arrows). (PDF) [file pone.0245417.s002.pdf]
