# Supplementary material for: The impact of COVID-19 vaccination campaigns accounting for antibody-dependent enhancement
Source: PLoS One. 2021 Apr 22;16(4):e0245417. doi: 10.1371/journal.pone.0245417 (PMC8061987; doi:10.1371/journal.pone.0245417)
Supplement: S2 Fig — Parameters for contact reduction are given in S7 Table. (PDF) [file pone.0245417.s003.pdf]

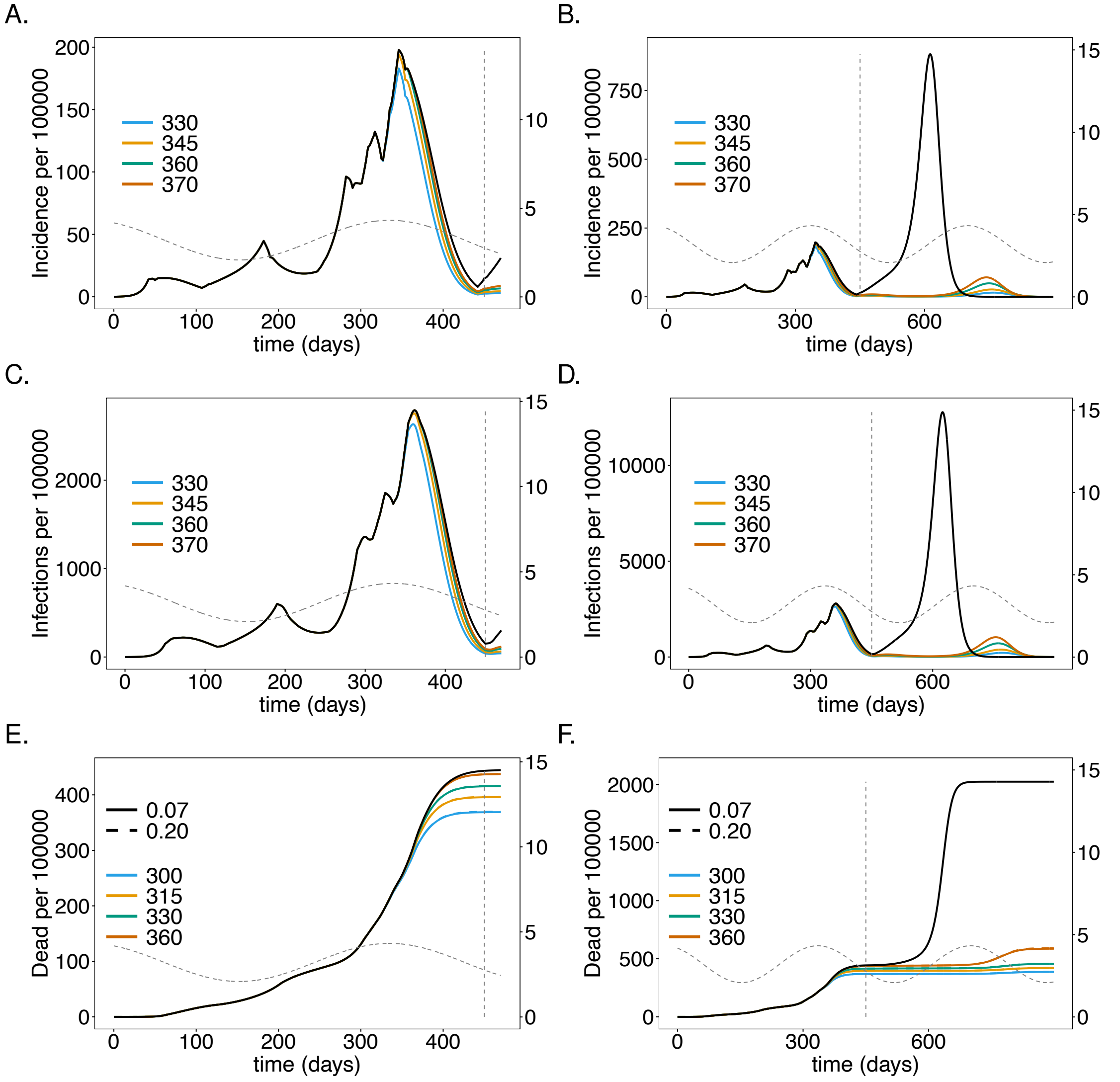

S2 Fig. Onset of the vaccination campaign: As in Fig 4 but for U.S. instead of Germany. Parameters for contact reduction are given in S7 Table.
