# Supplementary figures and images for: The impact of COVID-19 vaccination campaigns accounting for antibody-dependent enhancement
Source: PLoS One. 2021 Apr 22;16(4):e0245417. doi: 10.1371/journal.pone.0245417 (PMC8061987; doi:10.1371/journal.pone.0245417)

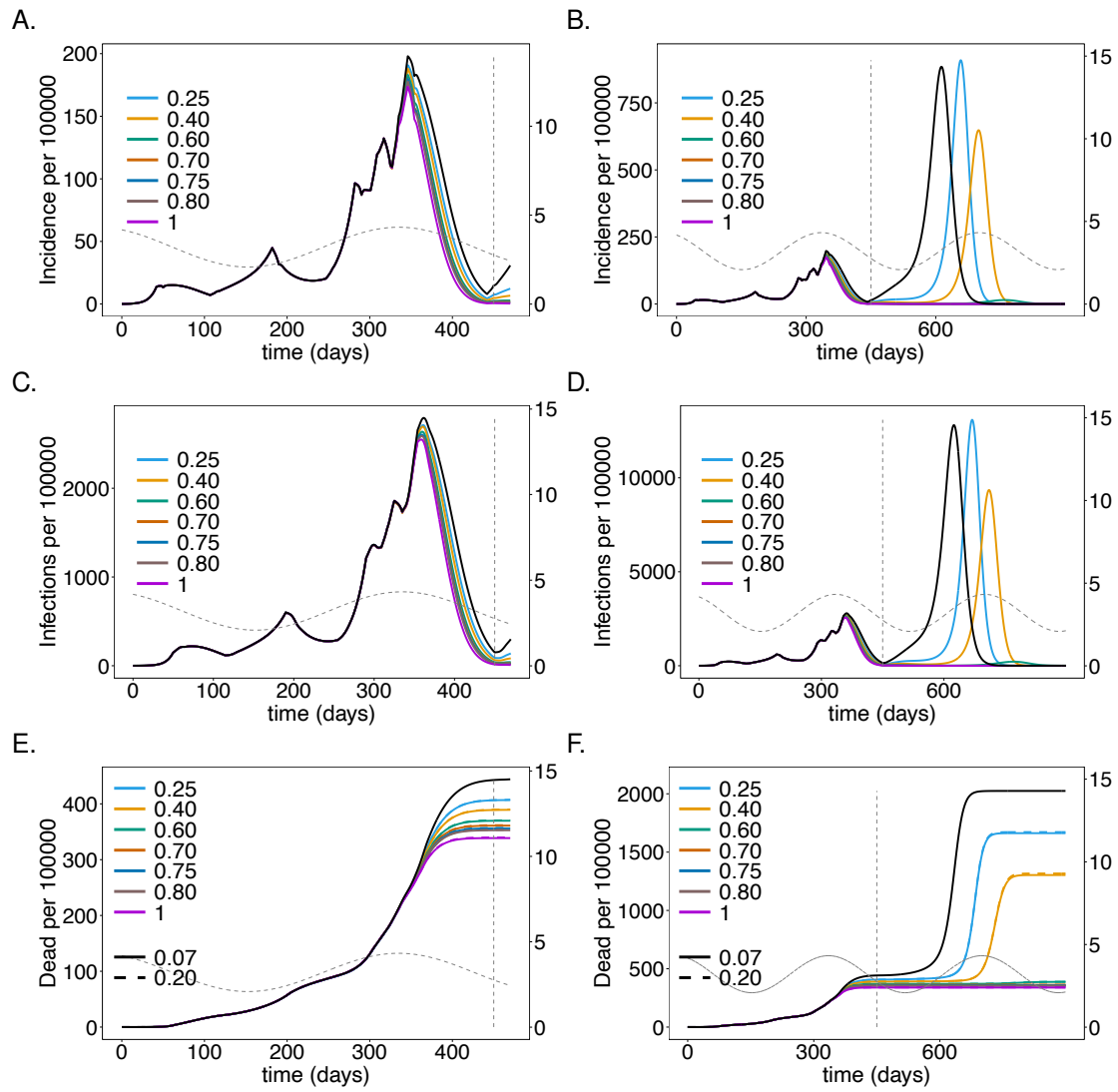

**S3 Fig. Vaccination coverage.** As Fig 5, but for the USA instead of Germany.

Supplement: S3 Fig — As Fig 5, but for the USA instead of Germany. (PDF) [file pone.0245417.s004.pdf]

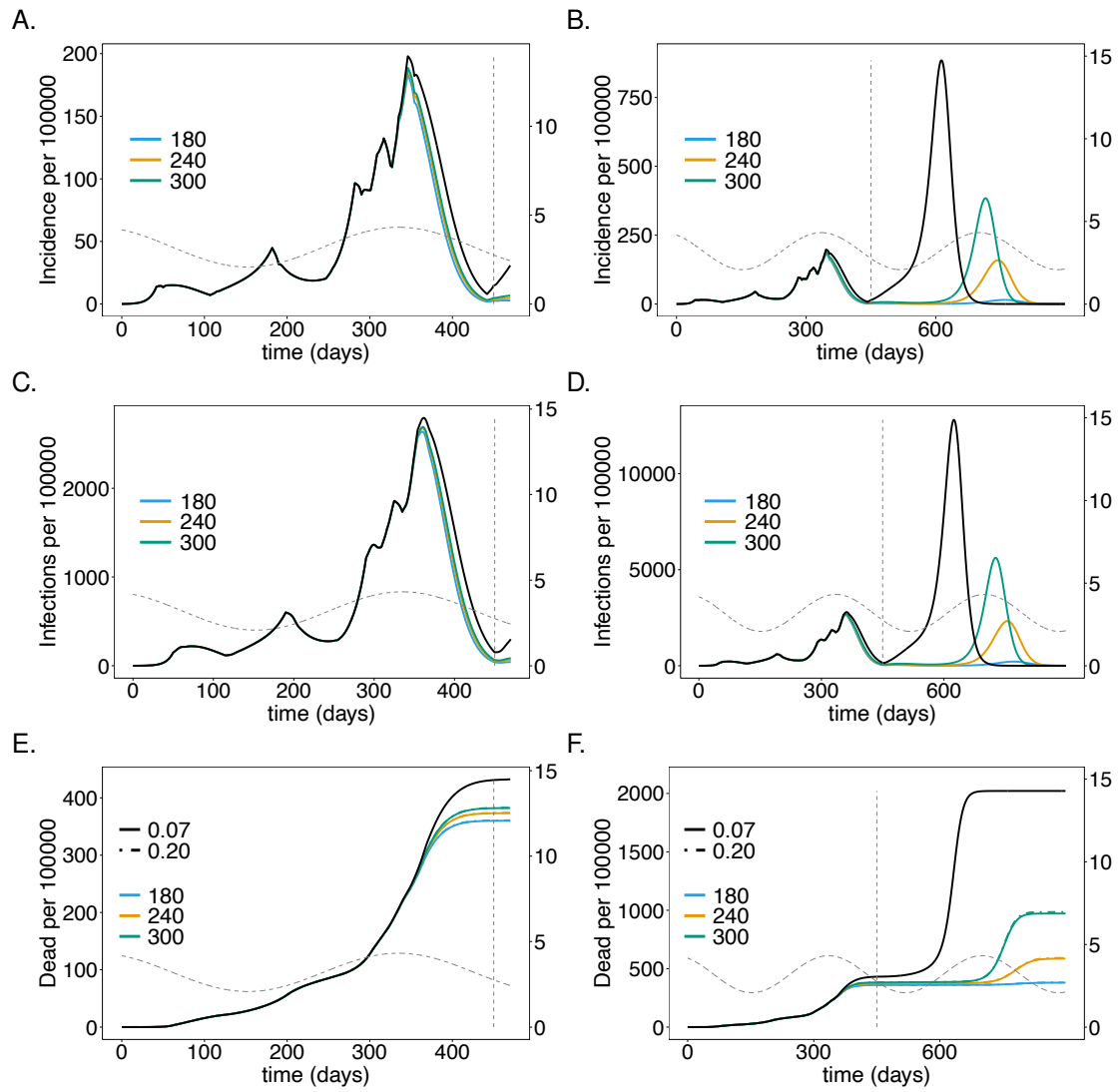

**S4 Fig. Vaccination rate.** As Fig 6, but for the USA instead of Germany.

Supplement: S4 Fig — As Fig 6, but for the USA instead of Germany. (PDF) [file pone.0245417.s005.pdf]

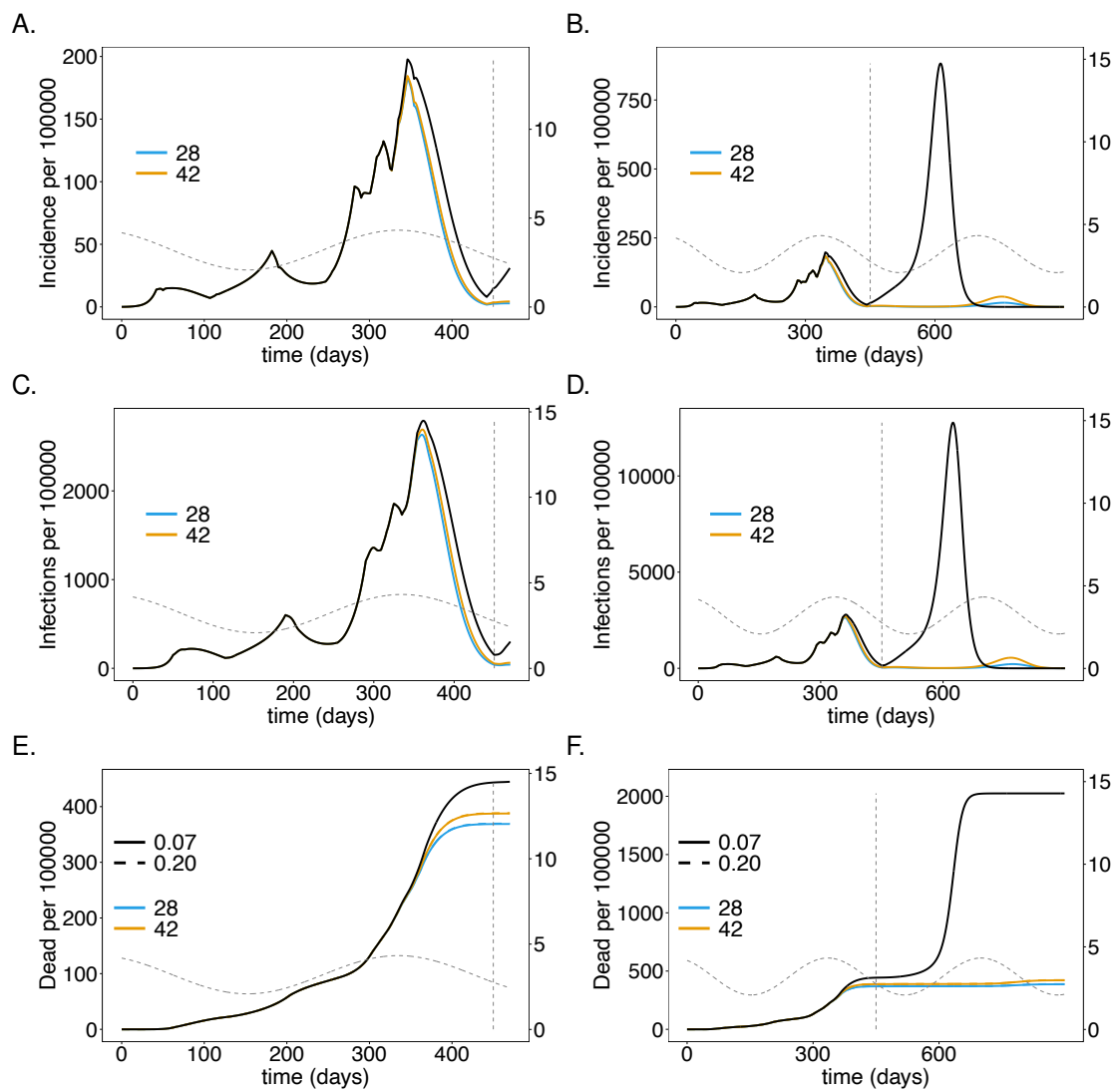

**S5 Fig. Vaccination schedule and time to immune response.** As Fig 7, but for the USA instead of Germany.

Supplement: S5 Fig — As Fig 7, but for the USA instead of Germany. (PDF) [file pone.0245417.s006.pdf]

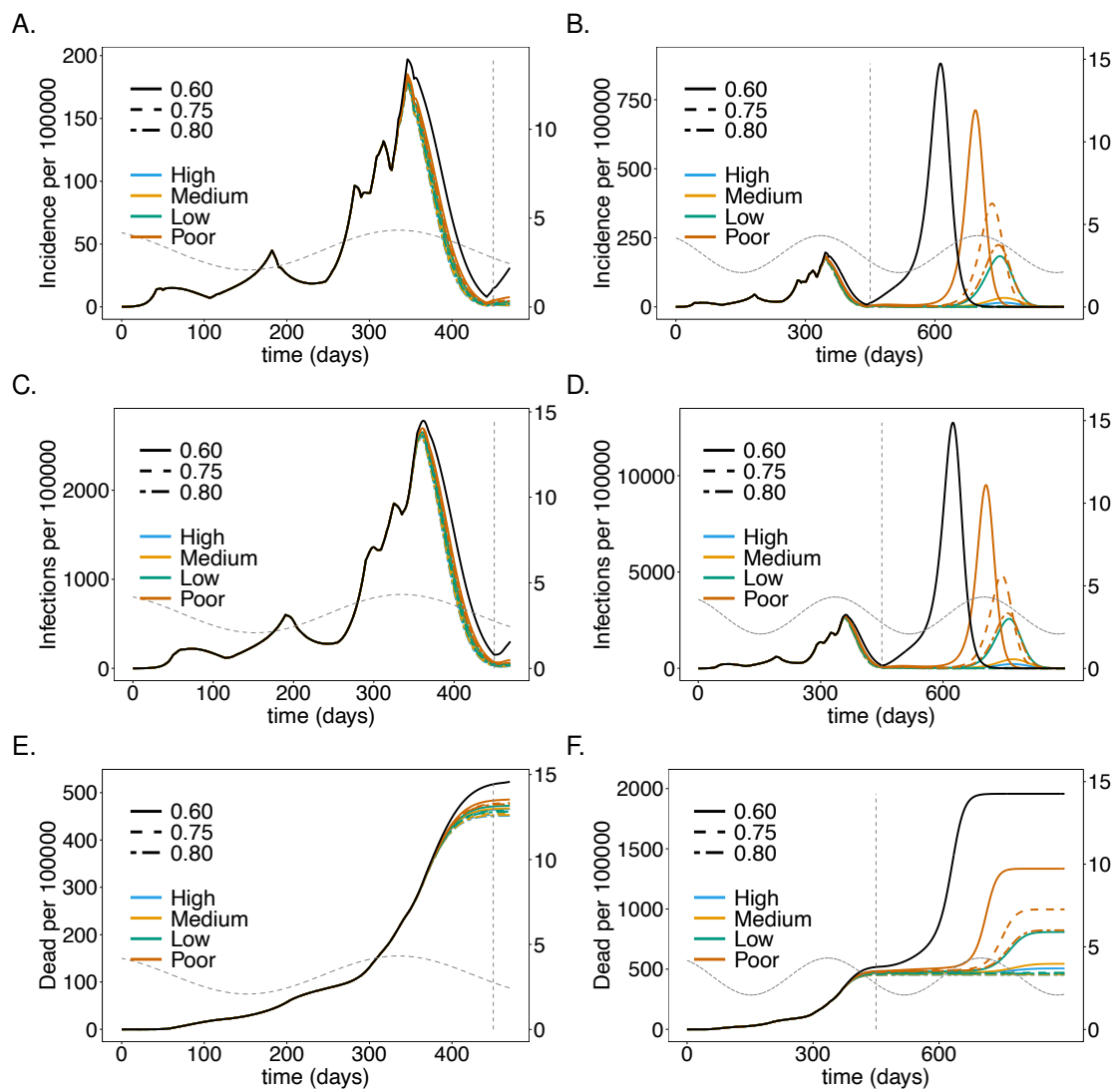

**S6 Fig. Vaccine effectiveness.** As Fig 8, but for the USA instead of Germany.

Supplement: S6 Fig — As Fig 8, but for the USA instead of Germany. (PDF) [file pone.0245417.s007.pdf]

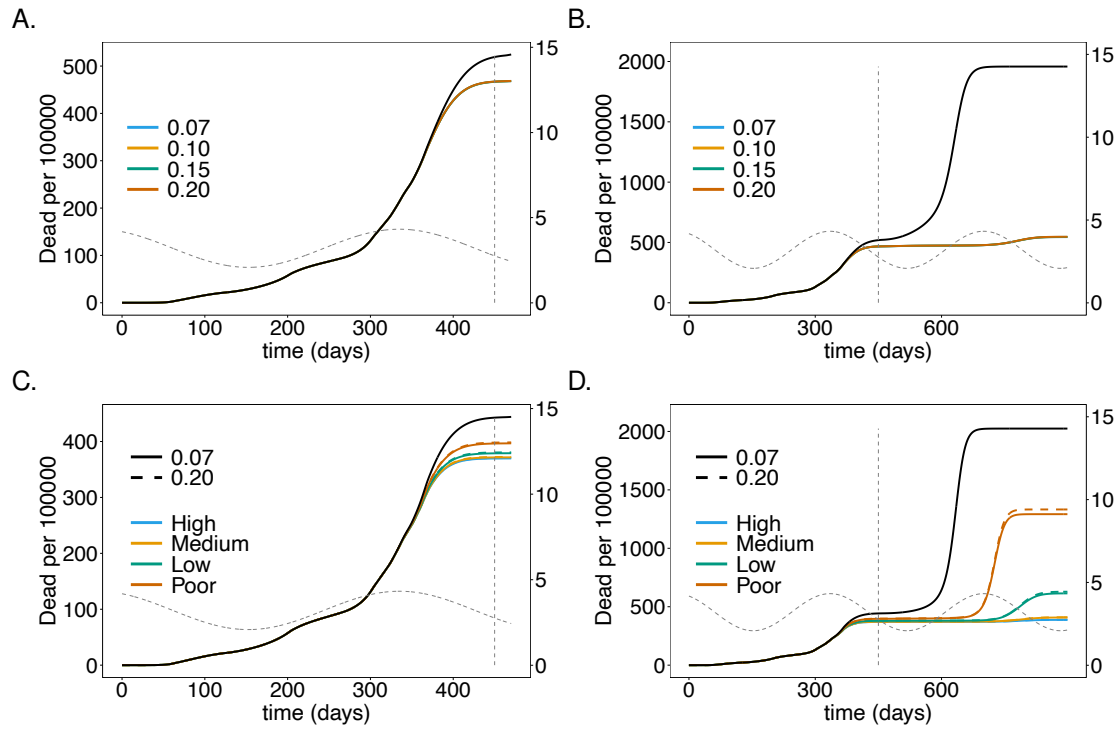

**S7 Fig. ADE-induced increased mortality.** As Fig 9, but for the USA instead of Germany.

Supplement: S7 Fig — As Fig 9, but for the USA instead of Germany. (PDF) [file pone.0245417.s008.pdf]
