# Supplementary material for: The impact of COVID-19 vaccination campaigns accounting for antibody-dependent enhancement
Source: PLoS One. 2021 Apr 22;16(4):e0245417. doi: 10.1371/journal.pone.0245417 (PMC8061987; doi:10.1371/journal.pone.0245417)
Supplement: S1 Table — (PDF) [file pone.0245417.s011.pdf]

S1 Table. (Sub-) population sizes of Germany (GER) and the USA chosen in simulations.

Parameter	Description	GER	USA
N	Total population size	83 000 000	331 000 000
$N^{(\mathrm{NV})}$	Size of unvaccinable population (40% of N)	33 200 000	132 400 000
$N^{(\mathrm{U})}$	No. of individuals waiting to be vaccinated ($N - N^{(\mathrm{NV})}$)	49 800 000	198 600 000
