# Supplementary material for: The impact of COVID-19 vaccination campaigns accounting for antibody-dependent enhancement
Source: PLoS One. 2021 Apr 22;16(4):e0245417. doi: 10.1371/journal.pone.0245417 (PMC8061987; doi:10.1371/journal.pone.0245417)
Supplement: S2 Table — (PDF) [file pone.0245417.s012.pdf]

S2 Table. Parameters describing disease progression for Germany (GER) and the USA.

Parameter	Description	Value
n_E	No. of latent sub-states (Erlang states)	16
n_P	No. of prodromal sub-states (Erlang states)	16
n_I	No. of fully infectious sub-states (Erlang states)	16
n_L	No. of late infectious sub-states (Erlang states)	16
D_E	Average duration of latent period	3.7 days
D_P	Average duration of prodromal period	1 day
D_I	Average duration of fully infectious period	5 days
D_L	Average duration of late infectious period	5 days
ε	Transition rate of latent sub-states	n_{E}/D_E
φ	Transition rate of prodromal sub-states	$n_{\mathrm{P}}/D_{\mathrm{P}}$
γ	Transition rate of fully infectious sub-states	$n_{\mathrm{I}}/D_{\mathrm{I}}$
δ	Transition rate of late infectious sub-states	n_L/D_L
α	Average waiting time for the outcome of the vaccine	$1/28, 1/42$
ν	Rate at which individuals get vaccinated	$0, 1/180, 1/240, 1/300$

Parameters describing disease progression and their values used in the simulations. Abbreviations: No. ... Number.
