# Supplementary material for: The impact of COVID-19 vaccination campaigns accounting for antibody-dependent enhancement
Source: PLoS One. 2021 Apr 22;16(4):e0245417. doi: 10.1371/journal.pone.0245417 (PMC8061987; doi:10.1371/journal.pone.0245417)
Supplement: S3 Table — (PDF) [file pone.0245417.s013.pdf]

S3 Table. Summary of variables describing sub-population sizes in Germany (GER) and the USA.

Name	Description	Initial Values GER	Initial Values USA
$S^{(\mathrm{NV})}(t)$	Unvaccinable susceptibles (anti-vaxxers & inds. that cannot be vaccinated)	33 200 000	132 400 000
$S^{(\mathrm{U})}(t)$	Susceptibles waiting to be vaccinated	49 799 800	198 599 925
$S^{(\mathrm{V})}(t)$	Vaccinated susceptibles with pending vaccine outcome		0
$S^{(\mathrm{NI})}(t)$	Vaccinated susceptibles that failed to immunize		0
$S^{(\mathrm{PI})}(t)$	Vaccinated susceptibles who developed partial immunity		0
$S^{(\mathrm{ADE})}(t)$	Vaccinated susceptibles who developed ADE		0
$E_k^{(\mathrm{U})}(t)$	Latently inf. inds. waiting to be vaccinated		0
$E_k^{(\mathrm{V})}(t)$	Vaccinated latently inf. inds. with pending vaccine outcome		0
$E_k^{(\mathrm{NI})}(t)$	Unvaccinable latently inf. inds. and vaccinated ones who failed to immunize		0
$E_k^{(\mathrm{PI})}(t)$	Vaccinated latently inf. inds. who developed partial immunity		0
$E_k^{(\mathrm{ADE})}(t)$	Vaccinated latently inf. inds. who developed ADE		0
$P_k^{(\mathrm{U})}(t)$	Vaccinated prodromal inds. waiting to be vaccinated		0
$P_k^{(\mathrm{V})}(t)$	Vaccinated prodromal inds. with pending vaccine outcome		0
$P_k^{(\mathrm{NI})}(t)$	Unvaccinable prodromal inds. and vaccinated ones who failed to immunize		0
$P_k^{(\mathrm{PI})}(t)$	Vaccinated prodromal inds. who developed partial immunity		0
$P_k^{(\mathrm{ADE})}(t)$	Vaccinated prodromal inds. who developed ADE		0
		GER	USA
$I_k^{(\mathrm{U},-)}(t)$	Undiagnosed and asymptomatic fully inf. inds. waiting to be vaccinated	$k=1$: 200	75
		$k>1$: 0	0
$I_k^{(\mathrm{U},+)}(t)$	Diagnosed or symptomatic fully inf. inds. that were waiting to get vaccinated		0
$I_k^{(\mathrm{V})}(t)$	Fully inf. inds. with pending vaccine outcome		0
$I_k^{(\mathrm{NI})}(t)$	Unvaccinable fully inf. inds. and vaccinated ones who failed to immunize		0
$I_k^{(\mathrm{PI})}(t)$	Vaccinated fully inf. inds. who developed partial immunity		0
$I_k^{(\mathrm{ADE})}(t)$	Vaccinated fully inf. inds. who developed ADE		0
$I_k^{(I,\mathrm{V})}(t)$	Fully inf. inds. who were vaccinated during this phase with pending vaccine outcome		0
$I_k^{(I,\sim)}$	Fully inf. inds. who were vaccinated during this phase and had a neutral outcome		0
$I_k^{(I,*)}$	Fully inf. inds. who were vaccinated during this phase and had a del. outcome		0
$L_k^{(\mathrm{U},+)}(t)$	Diagnosed or symptomatic late inf. inds. that were waiting to get vaccinated		0
$L_k^{(\mathrm{U},-)}(t)$	Undiagnosed and asymptomatic late inf. inds. waiting to be vaccinated		0
$L_k^{(\mathrm{V})}(t)$	Late inf. inds. with pending vaccine outcome		0
$L_k^{(\mathrm{NI})}(t)$	Unvaccinable late inf. inds. and vaccinated ones who failed to immunize		0
$L_k^{(\mathrm{PI})}(t)$	Vaccinated late inf. inds. who developed partial immunity		0
$L_k^{(\mathrm{ADE})}(t)$	Vaccinated late inf. inds. who developed ADE		0
$L_k^{(I,\mathrm{V})}(t)$	Late inf. inds. who were vaccinated while fully infectious with pending vaccine outcome		0
$L_k^{(I,\sim)}(t)$	Late inf. inds. who were vaccinated while fully infectious and had a neutral outcome		0
$L_k^{(I,*)}(t)$	Late inf. inds. who were vaccinated while fully infectious and had a del. outcome		0
$L_k^{(L,\mathrm{V})}(t)$	Late inf. inds. who were vaccinated during this phase with pending vaccine outcome		0
$L_k^{(L,\sim)}(t)$	Late inf. inds. who were vaccinated during this phase and had a neutral outcome		0
$L_k^{(L,*)}(t)$	Late inf. inds. who were vaccinated during this phase and had a del. outcome		0
$R(t)$	Recovered or fully immunized individuals		0
$D(t)$	Dead individuals		0

Summary of all model compartments and their initial values chosen for simulation. Abbreviations: del.... deleterious; inf. ... infectious; inds. ... individuals.
