# Supplementary material for: The impact of COVID-19 vaccination campaigns accounting for antibody-dependent enhancement
Source: PLoS One. 2021 Apr 22;16(4):e0245417. doi: 10.1371/journal.pone.0245417 (PMC8061987; doi:10.1371/journal.pone.0245417)
Supplement: S4 Table — (PDF) [file pone.0245417.s014.pdf]

S4 Table. Parameters describing disease severity and mortality for Germany (GER) and the USA.

Parameters	Description		Value
	Fraction of ...		
$f_S^{(\mathrm{NI})}$	...susceptibles that fails to immunize after vaccination		cf. Table 1
$f_S^{(\mathrm{PI})}$	...susceptibles that develops partial immunity after vaccination		-
$f_S^{(\mathrm{ADE})}$	...susceptibles that develops ADE after vaccination		-
$f_S^{(R)}$	...susceptibles that develops permanent immunity after vaccination		-
$f_E^{(\mathrm{NI})}$	...latently inf. that fails to immunize after vaccination		cf. Table 1
$f_E^{(\mathrm{PI})}$	...latently inf. inds. that develops partial immunity after vaccination		-
$f_E^{(\mathrm{ADE})}$	...latently inf. that develops ADE after vaccination		-
$f_E^{(R)}$	...latently inf. that develops permanent immunity		-
$f_P^{(\mathrm{NI})}$	...prodromal inds. that fails to immunize after vaccination		cf. Table 1
$f_P^{(\mathrm{PI})}$	...prodromal inds. that develops partial immunity after vaccination		-
$f_P^{(\mathrm{ADE})}$	...prodromal inds. that develops ADE after vaccination		-
$f_P^{(R)}$	...prodromal inds. that develops permanent immunity after vaccination		-
$f_I^{(\mathrm{U},+)}$	...detected or sympt. fully inf. inds. that were waiting for vaccination		0.005
$f_I^{(\mathrm{NI})}$	...fully inf. inds. that fails to immunize after vaccination		cf. Table 1
$f_I^{(\mathrm{PI})}$	...fully inf. inds. that develops partial immunity after vaccination		-
$f_I^{(\mathrm{ADE})}$	...fully inf. inds. that develops ADE after vaccination		-
$f_I^{(R)}$	...fully inf. inds. that develops permanent immunity after vaccination		-
$f_I^{(I,\sim)}$	...fully infectious inds. vaccinated during this phase with neutral outcome		0.95
$f_L^{(\mathrm{NI})}$	...late inf. inds. that fails to immunize after vaccination		0
$f_L^{(\mathrm{PI})}$	...late inf. inds. that develops partial immunity after vaccination		0
$f_L^{(\mathrm{ADE})}$	...late inf. inds. that develops ADE after vaccination		0
$f_L^{(R)}$	...late inf. inds. that develops permanent immunity after vaccination		0
$f_L^{(I,\sim)}$	...late infectious inds. vaccinated during the fully inf. phase with neutral outcome		0.95
$f_L^{(L,\sim)}$	...late infectious inds. vaccinated during this phase with neutral outcome		0.99
		GER	USA
f_{Sick}	...sympt. individuals	0.58	0.58
f_{Dead}	...sympt. inds. who die from COVID-19	0.016	0.04
f_{Iso}	...inds. who are isolated	0.58	0.48
$f_{\mathrm{Sick}}^{(\mathrm{PI})}$	...sympt. inds. with partial immunity		0.50
$f_{\mathrm{Dead}}^{(\mathrm{PI})}$	...sympt. inds. with partial immunity who die from COVID-19		0.02
$f_{\mathrm{Dead}}^{(\mathrm{ADE})}$	...sympt. inds. with ADE who die from COVID-19		0.07, 0.2
$f_{\mathrm{Sick}}^{(\mathrm{U},+)}$	...sympt. or diagnosed individuals that were waiting to be vaccinated		cf. eq. 3
$f_{\mathrm{Sick}}^{(\mathrm{ADE})}$	...sympt. inds. with ADE		0.92
$f_{\mathrm{Sick}}^{(I,*)}$	...sympt. fully inf. inds. vaccinated during this phase with del. outcome		0.58
$f_{\mathrm{Dead}}^{(I,*)}$	...lethal infs. among fully inf. inds. vaccinated during this phase with del. outcome		0.07
$f_{\mathrm{Sick}}^{(L,*)}$	...sympt. late inf. inds. vaccinated during this phase with del. outcome		0.58
$f_{\mathrm{Dead}}^{(L,*)}$	...lethal infs. among late inf. inds. vaccinated during this phase with del. outcome		0.07

Abbreviations: del. ...deleterious; eq. ...Equation; inf. ...infectious; infs. ...infections; inds. ...individuals; sympt. ...symptomatic.
