# Supplementary material for: The impact of COVID-19 vaccination campaigns accounting for antibody-dependent enhancement
Source: PLoS One. 2021 Apr 22;16(4):e0245417. doi: 10.1371/journal.pone.0245417 (PMC8061987; doi:10.1371/journal.pone.0245417)
Supplement: S5 Table — (PDF) [file pone.0245417.s015.pdf]

S5 Table. Parameters describing contact behavior and force of infection for Germany (GER) and the USA.

Parameter	Definition	Value/Eq.	
		GER	USA
λ_{Ext}	Infections from outside of the population	45/day	50/day
$\bar{R}_0$	Annual average basic reproduction number	3.4	3.2
a	Amplitude of the seasonal fluctuation in R_0	0.43	0.35
$t_{R_{0\max}}$	Day when R_0 reaches its maximum	300	335
$Q_{\max}$	Maximum capacity of isolation units per 10,000	200	30
t_{Iso_1}	Day case isolation measures start	30	20
t_{Iso_2}	Day case isolation measures end	900	900
p_{Home}	Contact reduction in home isolation		75%
c_P	Relative contagiousness in prodromal period		0.5
c_I	Relative contagiousness in fully inf. phase		1
c_L	Relative contagiousness in late inf. phase		0.5
$\beta_P(t)$	Seasonally varying effective contact rate of prodromal inds.		cf. eq. 7a
$\beta_I(t)$	Seasonally varying effective contact rate of fully infectious inds.		cf. eq. 7b
$\beta_L(t)$	Seasonally varying effective contact rate of late infectious inds.		cf. eq. 7c

Abbreviations: eq. . . . Equation; inf. . . . infectious; inds. . . . individuals.
