# Supplementary material for: The impact of COVID-19 vaccination campaigns accounting for antibody-dependent enhancement
Source: PLoS One. 2021 Apr 22;16(4):e0245417. doi: 10.1371/journal.pone.0245417 (PMC8061987; doi:10.1371/journal.pone.0245417)
Supplement: S6 Table — (PDF) [file pone.0245417.s016.pdf]

S6 Table. Contact reduction parameters chosen for the simulations of Germany.

Parameter	Description	Value
t_{Dist_1}	Day when first "hard lockdown" (general distancing) starts	40
t_{Dist_2}	Day when first "hard lockdown" ends and first "relief period" starts	82
t_{Dist_3}	Day when first "relief period" ends and "soft lockdown" starts	246
t_{Dist_4}	Day when "soft lockdown" ends and the second "hard lockdown" starts	280
t_{Dist_5}	Day when second "hard lockdown" ends and relief period starts	397
t_{Dist_6}	Day when general contact reduction ends	450
p_{Cont_1}	General contact reduction between individuals during the first lockdown	70%
p_{Cont_2}	General contact reduction during the first "relief period"	40%
p_{Cont_3}	General contact reduction during the "soft lockdown"	50%
p_{Cont_4}	General contact reduction between individuals during the "hard lockdown"	68%
p_{Cont_5}	General contact reduction between individuals during the second "relief period"	50%
