# Supplementary material for: The impact of COVID-19 vaccination campaigns accounting for antibody-dependent enhancement
Source: PLoS One. 2021 Apr 22;16(4):e0245417. doi: 10.1371/journal.pone.0245417 (PMC8061987; doi:10.1371/journal.pone.0245417)
Supplement: S7 Table — (PDF) [file pone.0245417.s017.pdf]

S7 Table. Contact reduction parameters chosen for the simulations of the USA.

Parameter	Description	Value
t_{Dist_1}	Day when first contact restrictions (e.g. travel bans etc.) start	50
t_{Dist_2}	Day when restrictions were partly lifted	115
t_{Dist_3}	Day when first "relief period" ends and a "hard lockdown" starts	190
t_{Dist_4}	Day when "hard lockdown" is relieved into a "soft lockdown"	255
t_{Dist_5}	Day when second "soft lockdown" ends and a new "hard lockdown" starts	290
t_{Dist_6}	Day when second "hard lockdown" ends and a "relief period" starts	309
t_{Dist_7}	Day when "relief period" ends and a subsequent "hard lockdown" starts	316
t_{Dist_8}	Day when "hard lockdown" ends and a stronger lockdown starts	325
t_{Dist_9}	Day when stronger "hard lockdown" ends and a "soft lockdown" starts	335
$t_{\mathrm{Dist}_{10}}$	Day when "soft lockdown" ends and "hard lockdown" starts	354
$t_{\mathrm{Dist}_{11}}$	Day when general contact reduction ends	450
p_{Cont_1}	General contact reduction between individuals during the first lockdown	55%
p_{Cont_2}	General contact reduction during the first "relief period"	22%
p_{Cont_3}	General contact reduction during the "hard lockdown"	55%
p_{Cont_4}	General contact reduction between individuals during the "soft lockdown"	45%
p_{Cont_5}	General contact reduction between individuals during the second "hard lockdown"	65%
p_{Cont_6}	General contact reduction between individuals during the second "relief period"	55%
p_{Cont_7}	General contact reduction during the "hard lockdown"	60%
p_{Cont_8}	General contact reduction during the stronger "hard lockdown"	70%
p_{Cont_9}	General contact reduction between individuals during the "soft lockdown"	55%
$p_{\mathrm{Cont}_{10}}$	General contact reduction between individuals during the "hard lockdown"	65%
