# Supplementary material for: The impact of COVID-19 vaccination campaigns accounting for antibody-dependent enhancement
Source: PLoS One. 2021 Apr 22;16(4):e0245417. doi: 10.1371/journal.pone.0245417 (PMC8061987; doi:10.1371/journal.pone.0245417)
Supplement: Attachment — Submitted filename: Responses to Reviewers.pdf [file pone.0245417.s019.pdf]

Responses to Review Comments for the manuscript with number [PONE-D-20-40700] - [EMID:8ba28b59c7a90f45], entitled "The impact of COVID-19 vaccination campaigns accounting for antibody-dependent enhancement"

Reviewers and suggested responses

Reviewer #1:

Comment 1: Vaccination is an important way to protect people against covid-19 and will help the world return to normality. In this study authors employed a complicated SEIR model to explore the impact of vaccination campaigns. A very detailed realisation of the infection phases and immunization effects (both lethal and beneficial) of vaccination is good and bad at the same time. It is good because it closely describes the realities; it is bad because it introduces too many model parameters most of which may be hard to estimate. With values of many model parameters unknown or having large variations, model predictions are problematic.

Response: This is a very important remark raised by the reviewer. Our objective is to provide a model for pandemic preparedness that facilitates decision-making regarding the vaccination strategy in the current pandemic. Hence, the focus is mechanistic modelling, which yields realistic dynamics, rather than estimating parameters. Of course, this is at a cost of model complexity involving a number of parameters. We want to point out that we refrained from developing a simplified SEIR model, which is mathematically convenient but yields unrealistic dynamics. We believe it is highly problematic to fit such simplified models to prevalence data. Namely, most SEIR models do not use Erlang states as we do to obtain realistic dynamics. As a consequence, exponentially distributed waiting times are modelled (as was explained in the paper), which yields unrealistic dynamics. Thus, model parameters will be biased and/or intuitive estimates need to be correct for the model bias before these are used in the model. Therefore, we intentionally decided on a realistic model, which can take inputs from intuitive estimates.
Our model parameters are by no means artificial convolutes or transformations of measurable quantities, but they are intuitive quantities. Some of these parameters are obvious, others can and should be estimated from incidence and clinical data, whereas others just cannot and - importantly - should not be estimated. Parameters such as the population size, the capacity of quarantine units, etc. are given facts. Parameters reflecting contact reductions were estimated from data on contact behavior. Past interventions are more or less known. Future lockdowns are impossible to predict, as these are political decisions. Other parameters such as the duration of the infectious stages should not be estimated from epidemiological data but clinical data. Estimates for these parameters exist. The effectiveness of vaccines and the duration until the vaccine manifests its effect should be estimated from phase III vaccine trials. Parameters such as the vaccination rate should not be estimated anyway, they should be determined based on the model predictions.
We exemplified how the model can be adjusted to a specific country, for which we chose Germany, where our group is centered. We made sure that our parameters reflect the situation in Germany by using appropriate estimates of the contact behavior, R0, seasonal fluctuations in R0, etc. available in the literature. This yielded accurate dynamics for the number of infections that occurred in Germany in the past year. Based on these predictions, we choose a range of values of parameters such as the waiting time to get vaccinated, or the proportion of the vaccinable population, which reflect the vaccination strategy. The model is intended to facilitate decision-making to identify proper vaccination strategies.
(i) We added a sentence in the abstract and introduction making clear right away, that the model is intended to facilitate decision-making regarding vaccination strategies. (ii) In the result section, where we introduce the model parameters, we added some lines that better describe how the

parameters were chosen. (iii) We added references that further explain the use of Erlang stages in our model, which are mainly responsible for the model's complexity. (iv) We added an appendix showing how the model can be adjusted to reflect the situation in the USA.

Comment 2: Although authors argued that the values of model parameters they used "reflect the situation in the Federal Republic of Germany. ", "The simulation results until late December (t = 300) match the disease incidence in Germany", it is hard to know how they obtained those values.

Response: We agree with the reviewer. Since most of the authors are based in Germany, most of the authors are very familiar with the contact reduction and parameters in Germany as we read about them daily. We agree that it is necessary for the reader to have more transparency on the parameter choices. As mentioned in the response to comment 1, we added statements in the result section explaining how some of the parameters were chosen, particularly those the model is most sensitive to, i.e., the contact reductions, which were indeed chosen based on estimates of the contact behavior. We did the same in the new appendix that shows predictions for the US.

Comment 3: In principle, it is possible to set another set of values which can also "match the disease incidence in Germany". To justify those values, one suggestion is to estimate the relevant model parameters using the data until December 2020.

Response: We understand the reviewer's argument. In practice, the most important characteristic is that the parameters have physical significance and that it is possible to determine their values from observed scenarios. The parameters set of values for the SEIR model yielding realistic dynamics might not be unique in theory - however, a completely different set of parameters yielding realistic dynamics will likely not have meaningful values. The model dynamics are most sensitive to the contact reductions imposed. These are given facts, and relatively easy to estimate. Other parameters such as the duration of the infectious stages are not as important as the model is not very sensitive to them. Parameters such as the external force of infection can be intuitively chosen for the initial phase, as these terms are insignificant once the number of infections grows exponentially. All parameter choices were not arbitrary but based on empirical evidence (cf. reply to comments 1 and 2).

Comment 4: In modelling of infectious diseases, sensitivity analysis is important to guarantee the prediction results. That is, how robust the model conclusions are under different combinations of model parameters.

Response: This is an important point raised by the reviewer. It is important to investigate how the model responds to change in parameters or combinations of parameters. In fact, our whole study is in essence a sensitivity analysis, because - apart from the model parameters that are clearly known or intuitive - we provide simulations for a range of parameter combinations that describe the vaccination strategy. Obviously, it is impossible to provide results on all combinations of model parameters, as the number of combinations in such a complex model is infeasible. We would like to remind the reviewers that we mentioned in the manuscript that the base model (the model without vaccination) is the published covidsim model. This model is well understood and the effects of the various model parameters were described in detail. Moreover, the interested reader can use the web simulation http://covidsim.eu to explore and observe how changes in parameters affect the result. The effects of the parameters specific to this model, which are not understood, and are meaningless to estimate (because they reflect the vaccination strategy) were thoroughly explored.

Comment 5: Another issue is about the cost-effectiveness. In Introduction, authors mentioned many productions of covid-19 vaccine and their costs. In view of cost-effectiveness, what is the best vaccination campaign?

Response: Highlighting the potential cost of a vaccination campaign is important as the reviewer has pointed out. Obviously, the best strategy is to start as early as possible and to vaccinate as fast as possible. However, this is impossible for all countries at the same time with limited supply. There's just no one-size-fits-all. Rather the model is intended to adapt it to a particular country in question and determine the effects of different approaches.

The number of vaccine doses available depends on the pre-orders of other countries. Reaching a herd immunity threshold is different across countries. Namely, this threshold depends on R0 in each country. R0 depends on extrinsic factors such as climate and UV radiation, but also on the "normal" contact behavior and the population density. So do seasonal fluctuations in R0. Moreover, the vaccinable population depends on the country's demographics. Countries with a large underaged population, like in many countries in Sub-Saharan Africa, cannot reach the herd-immunity threshold because the vaccines are not approved for a large proportion of the population (e.g., in Rwanda the pop < 15 is 40% of the population, and those 15-24 another 20%, while those 65+ are just 2.7%). Moreover, the financial means to purchase vaccines depend on the GDP, as does the return of investment - in terms of costs of lockdowns and damage to the economy. Cost-effectiveness is hence relative to a country's GDP. However, macroeconomic considerations are not independent of other countries. E.g., while vaccinating early might save the economy billions in the short term when vaccinating early and fast, such a strategy might backfire in the long-term if - as a consequence - other countries cannot vaccinate fast enough and ultimately have to devalue their currencies.

Our intention was to give some background information to readers about covid vaccines in general. We refrained from estimating costs for a number of reasons. (i) Vaccine costs depend on the number of competing approved vaccines and on supply and demand. E.g., Israel is undoubtedly extremely successful in vaccinating its population. However, they pay twice the price as the EU. (ii) Countries are not independent in purchasing vaccines. E.g., the European countries have a common purchasing and distribution strategy. (iii) The acceptance of the vaccine will also be country-specific. (iv) Because of the reasons mentioned above a macroeconomic assessment is difficult to begin with.

Summarizing, a cost assessment would be definitely interesting, but exceeds the scope of our work and also our expertise. We refer the reviewer to the penultimate paragraph of the introduction for the questions, our model aims to answer, and to the discussion for concluding remarks on how our model has addressed them.

Reviewer #2:

I find the manuscript presents a predictive model with high confidence specifically designed for mid-size population countries to prepare for COVID19 vaccination strategies. Such tools are of great importance to public health administrations so that they can plan strategies such as prepare the healthcare workers for more rigorous countrywide vaccine campaigns and also prepare for the availability of enough vaccine doses. The manuscript is very well written and only needs few corrections. Some of the comments are attached.

Response: We thank the reviewer for this comment. We are pleased that our manuscript's objectives have been understood and described as well organized.

Comment 1: On page no. 7 in the result section for "Fraction of the population being vaccinated," it says: "Mortality substantially decreases as the proportion of the population getting vaccinated increases (Figs 5C, D). The reduction occurs in a nonlinear fashion and shrinks with higher proportions of the population being vaccinated." Do you mean to cite Fig 5 (E, F) here?

Response: The reviewer is correct, we fixed it!

Comment 2: Although somewhat obvious, easy to interpret figures if the black line is described as no vaccination in legends.

Response: We understand the reviewer's argument. We were actually aware of it initially and decided not to put the black line (no vaccination) in the legend for the following reason. Listed in the legends are the numerical values of the parameters that are chosen (except for Figs 8 and 9), no vaccination does not correspond to a unique parameter - except for the vaccination rate. We tried to put it in the legend but found it aesthetically most displeasing. We are willing to add it if necessary but we believe it is better as it is. Anyhow, the figures were updated, so the parameters now reflect the actual situation in Germany, where the hard lockdown (initially assumed to last until the end of January - it was officially supposed to end by the beginning of January but became obvious from the simulations that it will be extended) was extended to March 7, which is the new official end of the hard lockdown after which phases of relaxation will start.

Comment 3: In figure panel no. 5,6,7,8, figure B and D [fig 5,6,7,8] assume that contact reduction is no longer sustained. This concept should be clarified in figure legends as well.

Response: We fully agree. To resolve this issue, we added a vertical dashed line to the figures to indicate the time when the contact reduction measures are lifted.

Comment 4: I had a little hard time distinguishing the colors. In my opinion, in graphs, the choice of darker colors in legends would be more appropriate, perhaps.

Response: We thank the reviewer for the comment. We use colorblind-friendly palettes for the plots and at the same time be aesthetically pleasing. We increased the thickness of the legends and tried to adjust the brightness. We hope they are better readable now.

Comment 5: For "hard lockdown," it is assumed that international travel is restricted or controlled, which is not practical for the long term in many similar size population countries. Was this variant considered in the model?

Response: It is not explicitly considered for the following reason. Air travels are strongly controlled since the pandemic. Typically tests before departure are mandatory and entry tests often in combination with mandatory home quarantine. Therefore, air travel can be safely neglected in such a model (maybe not for all countries). More important seem to be infections from outside the population by border crosses by car or train, which are less controlled, with no tests mandatory. In the model, these are taken into account (as is air travel) by the external force of infection. This term is most important only in the initial spread, or when incidence is close to 0. Once the disease spreads exponentially, these terms (that translate to linear terms in the dynamics) are negligible. We decided not to discuss this in detail in the ms because we feel it would create more confusion than benefit. At least for European countries mobility across the border by road transportation seems more relevant.

Reviewer #3:

The article is well written. The author used the extended model of the SEIR to predict the occurrence of ADE during vaccination campaigns in the ongoing COVID-19 pandemic. Several different vaccination schedules were assumed and their effects were analyzed using the extended SEIR model, including the influence of the onset of vaccination campaigns, the vaccination rate, the vaccination coverage, vaccination schedules, effectiveness of the vaccine, and adverse side effects, and so on. Also, the model was parameterized to reflect the situation in the Federal Republic of Germany that so far intervened successfully in the COVID-19 pandemic. The results matched the situation in Germany. But the applicability of the model to other countries needs to be considered. To some extent, this model can predict the effect of the vaccination campaign.

Response: We thank the reviewer for this comment. We are glad the manuscript is perceived as well-written and easy to understand. To keep the ms short and readable we just focused on Germany. However, we agree with the reviewer and added simulations reflecting the situation in the US as an appendix and describe the results in comparison to those for Germany. We tried several other countries but decided it would be more appropriate to present them in a follow-up article that focuses less on the model itself.

Minor comments
Comment 1: “invective” should be “infective” instead in Fig 1 legend.
Done.
Comment 2: “an” should be revised as “and” on line 178.
Done.
