# Supplementary material for: The impact of COVID-19 vaccination campaigns accounting for antibody-dependent enhancement
Source: PLoS One. 2021 Apr 22;16(4):e0245417. doi: 10.1371/journal.pone.0245417 (PMC8061987; doi:10.1371/journal.pone.0245417)
Supplement: Attachment — Submitted filename: CovidSiM2.0_ReviewComments_PJB.docx [file pone.0245417.s018.docx]

The current manuscript describes the predictive model and tool CovidSIM, Pandemic Preparedness Planning tool for COVID-19 in Germany. Compare to CovidSIM1.0 current model (CovidSIM2.1) takes into consideration the occurrence of ADE during vaccination campaigns. The population was partitioned into several groups, and groups were modeled into the SEIR model, assuming a closed population. Effect of vaccine, case isolation, and also general contact was modeled in. The authors take advantage of the additional co-variants in the model by adding vaccination campaigns and their effectiveness. The purpose of modeling was to illustrate the effects of vaccination campaigns, rate of vaccination, and principal effectiveness of vaccination for the severity of Antibody-dependent enhancement (ADE), which is one of the side effects of the vaccine.

The goal of the study overall was to explore how fast herd immunity can be reached with a vaccination campaign, considering that contact reductions are not a long-term solution. They predict that the vaccination coverage of 80% would result in high levels of herd immunity to allow a return to normality by summer 2021 in Germany.

I find the manuscript presents a predictive model with high confidence specifically designed for mid-size population countries to prepare for COVID19 vaccination strategies. Such tools are of great importance to public health administrations so that they can plan strategies such as prepare the healthcare workers for more rigorous countrywide vaccine campaigns and also prepare for the availability of enough vaccine doses. The manuscript is very well written and only needs few corrections. Some of the comments are below.

Minor comments:

- On page no. 7 in the result section for “Fraction of the population being vaccinated,” it says: “Mortality substantially decreases as the proportion of the population getting vaccinated increases (Figs 5C, D). The reduction occurs in a nonlinear fashion and shrinks with higher proportions of the population being vaccinated.” Do you mean to cite Fig 5 (E, F) here?
- Although somewhat obvious, easy to interpret figures if the black line is described as no-vaccination in legends.
- In figure panel no. 5,6,7,8, the figure B and D [ fig 5,6,7,8] assume that contact reduction is no longer sustained. This concept should be clarified in figure legends as well.
- I had a little hard time distinguishing the colors. In my opinion, in graphs, the choice of darker colors in legends would be more appropriate, perhaps.
- For “hard lockdown,” it is assumed that international travel is restricted or controlled, which is not practical for the long term in many similar size population countries. Was this variant considered in the model?
